# Perioperative and Follow-Up Analyses of Primary Posterior Stabilized and Cruciate Retaining Knee Arthroplasty

**DOI:** 10.3390/jcm14113752

**Published:** 2025-05-27

**Authors:** Isabel Reckermann, Patrick Orth, Christian Götze, Filippo Migliorini, Cueneyt Sönmez, Julian Koettnitz

**Affiliations:** 1 Faculty of Medicine, Ruhr-University Bochum, Universitätsstraße 150, 44801 Bochum, Germany; isabelreckermann@gmx.de (I.R.); patrick.orth@muehlenkreiskliniken.de (P.O.); christian.goetze@sfh-muenster.de (C.G.); cueneyt.soenmez@muehlenkreiskliniken.de (C.S.); 2 Department of General Orthopaedics, Auguste-Viktoria-Clinic Bad Oeynhausen, University Hospital of RUB-Bochum, Am Kokturkanal, 32545 Bad Oeynhausen, Germany; 3 Department of Orthopaedics and Trauma Surgery, University Clinic Aachen, RWTH Aachen University Clinic, 52064 Aachen, Germany; migliorini.md@gmail.com

**Keywords:** age, cruciate retaining, gender, posterior stabilized, total knee arthroplasty

## Abstract

**Background:** Total knee arthroplasty (TKA) is a widely performed procedure to alleviate pain and restore function in patients with advanced knee osteoarthritis. Two common implant designs are cruciate-retaining (CR) and posterior-stabilized (PS) knees. Despite extensive research, the superiority of one design over the other remains inconclusive. **Methods:** A prospective analysis was conducted on 123 patients who underwent total knee arthroplasty (TKA) between June 2022 and June 2023 at a university hospital. Demographic data, mobility, the use of walking aids, pre- and postoperative range of motion and leg axis as well as surgical and systemic complications were collected and compared between CR and PS-TKA. **Results:** The mean age of the patients was 67.94 ± 10.14 years and 65.9% were women. The time of operation was significantly different between PS- and CR-TKA (PS: 83.31 ± 25.65 min; CR: 95.26 ± 24.61 min; *p* = 0.011). The pre- to postoperative leg axis after six months was significantly different in both groups (PS: 7.06° ± 4.76°; CR: 6.25° ± 3.13°; *p* = 0.001). The range of motion (ROM) (PS: 105.19° ± 15.56°; CR: 93.29° ± 15.09°; *p* = 0.001) as well as the deficit after six months (PS: 23.56° ± 19.73°; CR: 37.57° ± 23.33°; *p* = 0.003) between patients with PS and CR-TKA were significantly different. Gender (male vs. female PS/CR) and age (<75 years vs. >75 years PS/CR) differences were shown for the ROM and flexion deficit after six months (*p* = 0.003; *p* = 0.005). For age, a significant difference was shown for the quality of life (mean ranks: <75 y: 47.96; >75 y: 31.03; *p* = 0.009) and WOMAC score (mean ranks: <75 y: 38.27; >75 y: 61.75; *p* = 0.001) after six months. **Conclusions:** This study shows the different outcomes for posterior-stabilized versus cruciate-retaining TKA with regard to time of surgery, range of motion, and flexion deficit after 6 months with PS-TKA yielding better results. The gender analyses revealed similar outcomes after six months between both arthroplasty groups, whereas the age analyses revealed significant differences. The standardized use of PS-TKA for the elderly is recommended.

## 1. Introduction

Total knee arthroplasty (TKA) is a well-established procedure for the treatment of end-stage knee arthritis, with both posterior-stabilized (PS) and cruciate-retaining (CR) designs widely used in clinical practice. For example, the predicted total annual counts for primary TKA in the United States in 2030 and 2040 will rise from about 1.5 million to about 3.5 million procedures per year, respectively [1]. Thereby, TKAs are mainly implanted using the cemented technique. From the Swedish, United Kingdom (UK), Australian, and United States (U.S.) registries, 62% to 91% of the arthroplasties were reported as “cemented”. Worldwide, in 2020, around 654 million individuals aged 40 years and older were estimated to suffer from osteoarthritis [2,3]. In patients with an intact posterior cruciate ligament (PCL), the choice between these two fundamental design philosophies continues to generate a significant debate among orthopedic surgeons regarding their comparative advantages in functional outcomes and long-term survivorship [4]. The PS design was developed to address potential issues with the PCL in TKA, providing stabilization through a cam-post mechanism that substitutes for the PCL function. In contrast, CR designs preserve the native PCL, potentially maintaining more natural knee kinematics. Each approach offers distinct theoretical benefits, with PS designs potentially allowing for greater flexion and stability, while CR designs may better preserve proprioception and require less bone resection [5]. Age-specific considerations have emerged as important factors in implant selection. A 2023 propensity score-matched cohort study comparing CR and PS designs found that PS implants demonstrated superior outcomes in the activities of daily living subscales compared with CR designs, leading researchers to suggest that PS designs may be preferable for elderly patients. This finding is particularly relevant given that the age of patients receiving TKAs is steadily increasing [6,7,8]. Studies investigating gender differences in primary knee arthroplasty often show a disadvantage for women. Differences have primarily been found in implant sizes, postoperative axis correction, and in the occurrence of complications, mobility, and outcome [9,10]. On the other hand, a prospective long-term study by Ayers et al. (2024) [11] revealed no further differences after 5 years. Although female patients had poorer preoperative scores, the postoperative scores were similar to those of men [11,12]. The issue of gender differences in primary knee arthroplasty therefore remains controversial. The primary objective of this study was to compare perioperative and short-term postoperative outcomes between posterior-stabilized (PS) and cruciate-retaining (CR) total knee arthroplasties (TKAs) in patients with advanced knee osteoarthritis. Specifically, the study aimed to analyze differences in mobility, range of motion, leg axis correction, and pain between the two implant designs at six months postoperatively. Additionally, the study sought to investigate the impact of patient gender and age on postoperative results including functional outcomes, quality of life, and patient satisfaction. By examining these variables in a prospective cohort, the study intends to provide evidence to guide implant selection and optimize clinical decision-making for diverse patient populations undergoing primary TKA.

## 2. Methods

### 2.1. Study Design

The present study was performed according to Strengthening the Reporting of Observational Studies in Epidemiology (STROBE) [13]. The study was conducted in accordance with the Declaration of Helsinki and approved by the local Ethics Committee of the university hospital.

**Figure 1:**
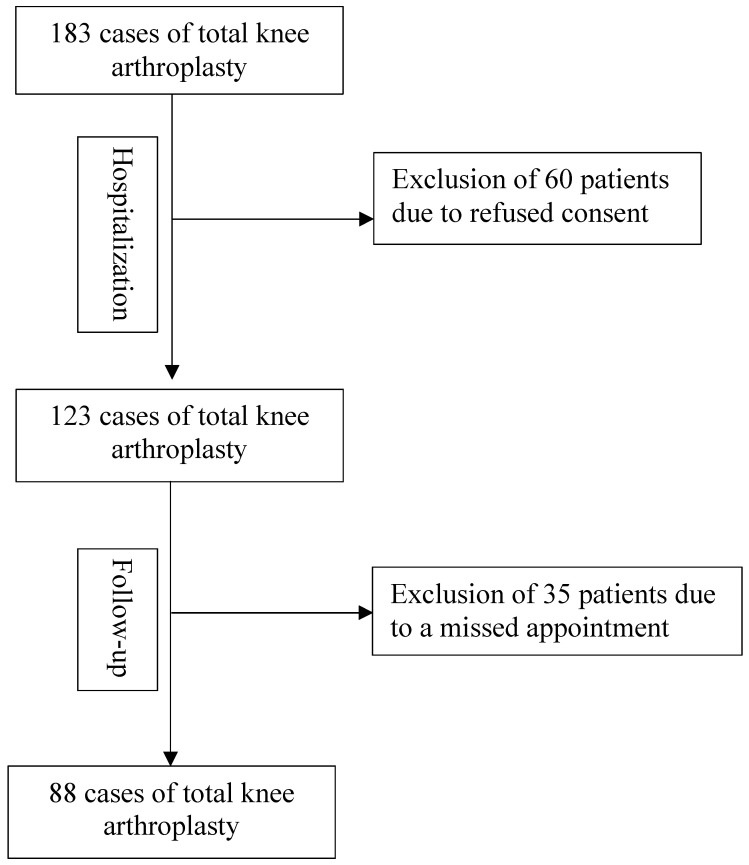
Flowchart study design.

Data from patients who underwent primary total knee arthroplasty between June 2022 and June 2023 were retrieved. A consecutive data collection was provided during the hospitalization, and a prospective follow-up investigation was conducted after six months (Figure 1). The data were retrieved using Pegasos 7 (Nexus Marabu GmbH, Berlin, Germany) and collected in Microsoft Excel (Microsoft Corporation, Redmond, WA, USA). The initial variables to be examined yielded a higher sample size with an alpha level of 0.05 and a test power of 80% for three predictors (prosthesis types, gender, age) in the a priori power analysis. Significantly fewer variables were ultimately examined, which meant that both the perioperative and postoperative data analysis after 6 months yielded a sufficient number of patients. The following data were collected at admission: age, sex, side, body mass index (BMI), length of hospital stay, length of intensive care unit stay, the number of pre-diseases (represented by the median), and American Society of Anesthesiologists physical status (ASA). The ASA classification is rated from 1 to 6 (normal health, mild, severe, severe with life-threatening conditions, moribund diseases, and brain dead) [14]. The following data were collected during the hospitalization: total knee replacement, preoperative mobility, preoperative and postoperative axis, serological data (preoperative and postoperative hemoglobin, C-reactive protein (CRP), sodium, potassium, international normalized ratio (INR), the incidence of systemic and surgical complications, and the frequency of blood unit transfusions. Systemic complications included pulmonary, cardiac, urogenital, and neurologic complications. Surgical-related complications included early infections, neurologic disorders, fractures, bleeding, aseptic loosening, surgical interventions post-surgery, and post-discharge complications such as infections or instabilities. After six months, the valgus/varus axis, the range of motion, occurred systemic and surgical complications, the quality of life, and the patient satisfaction with the Western Ontario and McMaster Universities Osteoarthritis Index (WOMAC) score were collected.

### 2.2. Exclusion Criteria

If patient data were not accessible, the patient was excluded from the present investigation.

Patients with aseptic and septic revision knee arthroplasty were excluded, as were patients with a primary rotational knee arthroplasty and patients with missing or refused consent.

### 2.3. Inclusion Criteria

All patients undergoing primary total knee arthroplasty were retrieved, and their eligibility was assessed. The inclusion criteria were: (1) patients with primary and secondary arthrosis (2) patients aged between 20 and 90 years; (3) accessible patient data; (4) patients with enrolled informed consent.

### 2.4. Perioperative Management

The total knee arthroplasties were conducted by applying the Smith and Nephew Genesis II CR/PS and Journey II CR/BCS system. The selection of the prothesis design Genesis II and Journey II was the result of many years of using the implants as the “in-house standard” at our surgical center with access to long-term results. In addition, comparability was ensured by using a single manufacturer for all implants, and the implants are also used globally. A medial parapatellar approach was used for all cases. The mechanical alignment strategy was used for all TKAs examined. This involved aligning the implant at a 90° angle to the mechanical axes. All patients received general anesthesia and at least short-term monitoring after the operation. A stationary or ambulant rehabilitation program was organized prior to hospital release.

### 2.5. Blood Unit Supply

The indication for the blood unit transfusion was according to the restrictive Cochrane guidelines: Hb-levels over 8.0 g/dL indicated no transfusion; between 7 g/dL and 9 g/dL with concomitant clinical symptoms such as dizziness, nausea, malaise, or loss of appetite; and Hb-levels under 8 g/dL indicated transfusion [15].

### 2.6. Appointment After Six Months

Enrollment required a signed informed consent information. Patients were examined by the same person, who collected the perioperative data. The six-month routine check-ups were carried out by the university health center. The full leg standing X-ray was performed as the standard X-ray examination. The patient was asked to bring all possible data from the rehabilitation center. Furthermore the patients were asked for their satisfaction (1–5, no satisfaction to completely satisfied), quality of life (QoF) (1–5, significantly poorer QoF to significantly improved QoF), and WOMAC-score (pain assessment, stiffness, difficulty assessment) [16].

### 2.7. Statistical Analyses

All statistical analyses were performed using the software IBM SPSS version 29 (IBM, Armonk, NY, USA). Metric-scaled data were analyzed by the mean, standard deviation, and variance. Nominal, dichotomous data were analyzed by Fisher’s exact test. For the analysis of metric and nominally scaled variables, the T-test for independent samples, variance analyses, the Levene test, and the Welch tests were used. Cohen’s d (small 0.20; medium 0.50; large 0.80) and the 5% interval were used as effect sizes. The effect size used was phi (small 0.10; medium 0.30; large 0.50). For correlation analyses of the metrical and ordinal scales, the bivariate correlation Pearson and Spearman analyses were used. For nominal and ordinal scales, the Mann–Whitney U test and Kruskal–Wallis H test (KWH) were used. For nominal scales with more than two values and two different time points, a linear model with repetition of the measured values was used. For estimating the differences in the pain scale between the groups, we used the analysis of covariance (ANCOVA) to adjust the baseline differences in the pain scores and provide estimates for identifying differences between the groups. For radiological analyses of the axis of the knees, the mechanical leg angle was measured between the mechanical axis of the femur and the mechanical axis of the tibia with a fixpoint of 0° [17]. The range of motion was measured as a total value of the flexion and extension of the patient. The significance level was set two-sided with α = 0.05. The Bonferroni post hoc test was used for groups n = ≥3 and multiple testing to control the *p* values for their actual quality and relevance. Therefore, the alpha level was divided through the number of tests, which set the threshold to 0.0031.

## 3. Results

### 3.1. Recruitment Process

In total, data from 123 patients with primary TKA and a signed consent information were retrieved from June 2022 to June 2023. Data of all 123 patients were retrieved during hospitalization, and the data of 88 patients were collected in the follow-up.

### 3.2. Patient Demographics

The indications for total knee arthroplasty were 89.4% primary (110 of 123) and 10.6% (13 of 123) secondary knee arthrosis. A total of 18.7% (23 of 123) operations were carried out by residents, 100 (81.3%) operations were performed by a specialist. Overall, the posterior cruciate ligament was resected in 58.5% (72 out of 123) and preserved in 41.4% (51 out of 123) of the cases. More demographic data is shown at Table 1.

### 3.3. Mobility

The preoperative, postoperative, and reappointment walking distance and the use of crushes were not significantly different between the PS and CR arthroplasties. For more details, see Table 2 and Table 3.

### 3.4. Leg Axis Measurement

**Figure 2:**
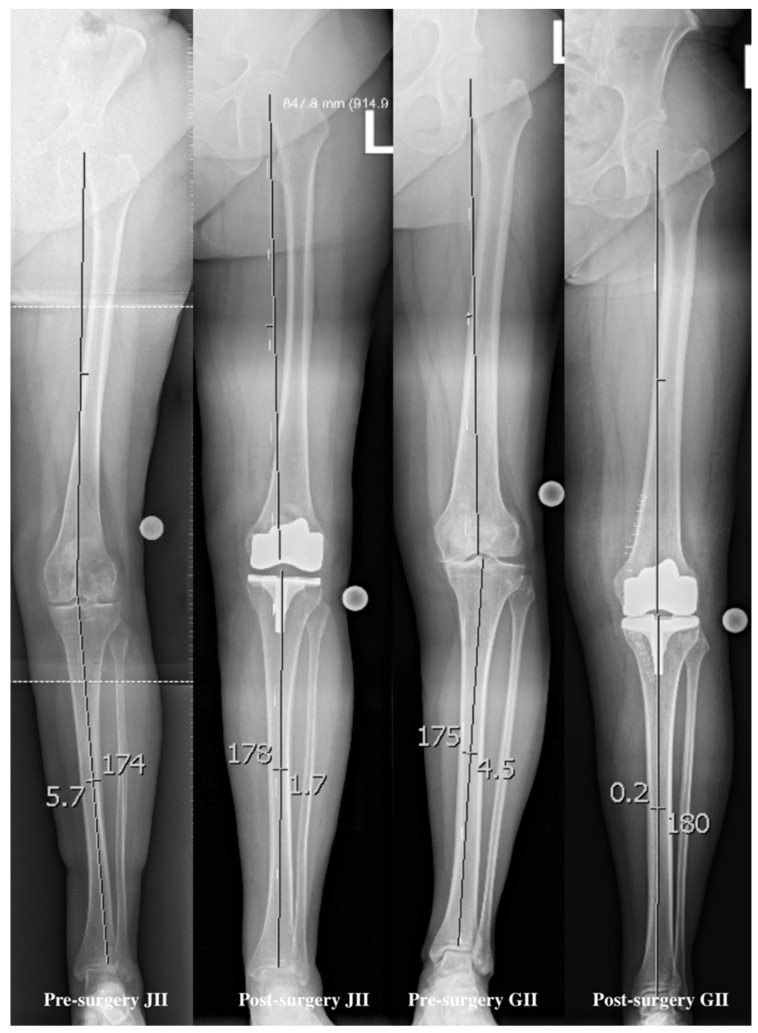
Examples of angle measuring, pre- and postoperatively, with the JII and GII arthroplasty. The fixed points were the hip joint center of rotation, the distal femoral center, the proximal tibial center, and the distal tibial center.

The degree correction of the axial misalignments (alpha-angle) was measured before and after, based on the work of Poilvache et al. (1996) [17]. Overall, the preoperative axis was significantly different to the postoperative axis and the axis after six months in the control (*p* = 0.001, 95%CI = 3.8021/6.0966; *p* = 0.001, 95%CI = 3.9005/6.2514), but no difference was found between the postoperative control and the six month control of the axis (*p* = 0.444, 95%CI = −0.6389/0.2831) (see Figure 2 for details). In general, patients with a higher postoperative axis were more satisfied with the arthroplasty after 6 months (*p* = 0.039, 95%CI = 0.006/0.435).

### 3.5. Range of Motion

For the range of motion, extension deficits, flexion deficits, and correction of the axis between the posterior-stabilized and cruciate-retaining knees, see Table 4.

### 3.6. Clinical Data

The operation time was significantly different between the CR and PS arthroplasties. The measurement of the numeric pain scale (1 to 10) in rest and movement showed similar values between the patients with PS- and CR-knees preoperative and after six months. No significant difference between the CR and PS groups was found in the degree of pain reduction (dpr). Furthermore, the ANCOVA test revealed no significant differences for the NRS values between the PS and CR groups (Table 5).

Overall, there was a significant pain reduction between the preoperative and postoperative scales after six months for all patients (difference pre-6 mo = rest: 2.72 ± 2.72; movement = 4.91 ± 2.99; for both *p* = 0.001). The preoperative high pain values at rest resulted in significantly worse outcomes in the quality of life and in the WOMAC score, but not in the satisfaction of the patient after six months (*p* = 0.032, 95%CI = −0.485/−0.017; *p* = 0.185/0.607; *p* = 0.069, 95%CI = −0.452/0.025). Patient satisfaction, quality of life, and the WOMAC score after six months were not significantly different between the PS and CR knees (*p* = 0.683, Z = −0.408; *p* = 0.672, Z = −0.423, *p* = 0.936, Z = −0.080).

### 3.7. Blood Parameters

The hemoglobin levels (measured in g/dL) before operation were significantly different between patients with PS and CR knee arthroplasties (mean = 13.94 ± 1.42/13.35 ± 1.18; *p* = 0.017, 95%CI = 1.067/10.795), but no differences were found before discharge (mean = 9.88 ± 1.38/9.29 ± 1.08; *p* = 0.061, 95%CI = −0.2743/12.0225). The CRP-levels (measured in mg/L) before discharge from the hospital were significantly different between the PS and CR knee patients (mean = 96.57 ± 65.38/69.43 ± 36.87; *p* = 0.006, 95%CI = 8.0814/46.1902).

### 3.8. Gender Analyses

A total of 25.2% (31 out of 123) of the male patients and 32.5% (40 out of 123) of the female patients received a posterior-stabilized (PS) knee arthroplasty, 8.9% (11 out of 123) of the male patients and 32.5% (40 out of 123) of the female patients received a cruciate-retaining arthroplasty, and 22.7% (20 of 88) of the male patients and 36.4% (31 of 88) of the female patients with a PS-knee arthroplasty and 8.0% (7 of 88) of the male patients as well as 31.8% (28 of 88) of the female patients attended the follow-up. For the BMI, postoperative hospitalization and the NRS pain scale pre- and postoperative after six months in rest and movement showed no significant difference (*p* = 0.375, 95%CI = 0.000/0.083; *p* = 0.145, 95%CI = 0.000/0.116; *p* = 0.064, 95%CI = 0.000/0.183; *p* = 0.539, 95%CI = 0.000/0.081; *p* = 0.533, 95%CI = 0.000/0.092; *p* = 0.885, 95%CI = 0.000/0.093. The operation time differed significantly between the arthroplasty groups in terms of gender (*p* = 0.007, 95%CI = 0.008/0.189). CR arthroplasty took a significantly longer time for men and women than the PS arthroplasty. Most significant were the time differences between the PS male and CR male (*p* = 0.048, 95%CI = −49.57/−0.11) and between the PS female and CR male (*p* = 0.011, 95%CI = 4.94/52.92). The total range of motion, flexion, and extension deficits did not differ preoperatively and during rehabilitation. After six months, a significant difference occurred for the range of motion and flexion deficit between the groups. In particular, CR knees showed a greater flexion deficit than the PS knees. No significant difference was found for surgery-related or systemic complications during hospitalization, and pre- and postoperative (six months) walking distance (*p* = 0.076, KWH = 6.880; *p* = 0.608, KWH = 1.832), pre- and postoperative use (after six months) of aids (*p* = 0.496, phi = 0.327; *p* = 0.992, KWH = 0.091), quality of life, satisfaction after surgery, and WOMAC score after 6 months (*p* = 0.413, KWH = 2.864; *p* = 0.204, KWH = 4.593; *p* = 0.993, KWH = 0.089) were also not significantly different (for more details see Table 6).

### 3.9. Age Analyses

Two age groups were formed: under 75 and over 75 years of age. A total of 45.5% (56 out of 123) of the patients under 75 years and 12.2% (15 out of 123) of the over 75 year old patients received a posterior-stabilized (PS) knee arthroplasty; 34.1% (42 out of 123) of the under 75 years old patients and 7.3% (9 out of 123) of the over 75 years old patients received a cruciate-retaining arthroplasty; 44.3% (39 out of 88) of the under 75 years old and 14.8% (14 of 88) of the over 75 years old patients who received a PS-knee arthroplasty and 34.1% (30 of 88) of the under 75 years old patients as well as 5.7% (5 of 88) of the over 75 years old patients who received a CR-knee arthroplasty attended the follow-up. For the BMI, the ASA score, duration of the surgery, the preoperative use of walking aids and their use after six months, and the preoperative walking distance, significant differences were found (*p* = 0.001, 95%CI = 3.796/7.920; *p* = 0.002, Z = −3.151; *p* = 0.006, 95%CI = 4.141/22.381; *p* = 0.001, phi = 0.351; *p* = 0.001, phi = 0.469; *p* = 0.004, Z = −2.871). For the NRS pain scale preoperative in rest, movement, and in rest after six months, surgical and systemic complications, and walking distance after 6 months, no significant differences were shown (*p* = 0.844, 95%CI = −1.343/1.101; *p* = 0.795, 95%CI = −0.915/0.703; *p* = 0.736, 95%CI = −1.246/0.884; *p* = 0.765, 95%CI = −0.255/0.346; *p* = 0.545, 95%CI = −0.203/0.382; *p* = 0.233, KWH = 4227). The NRS in movement after six months was significantly different for the two age groups (*p* = 0.049 95%CI = −1.051/0.003), but a closer view divided into PS and CR groups for both ages found no significant difference. The total range of motion, flexion, and extension deficits did not differ preoperatively and after six months for the age groups (*p* = 0.175, 95%CI = −2439/13,222; *p* = 0.270, 95%CI = −10,242/2887; *p* = 0.198, 95%CI = −4851/1016; *p* = 0.834, 95%CI = −7832/9689; *p* = 0.635, 95%CI = −14,603/8952; *p* = 0.771, 95%CI = −4476/3330).

A significant difference was found for the quality of life (mean ranks: <75 y: 47.96; >75 y: 31.03; *p* = 0.009, KWH = −2.610) and WOMAC score after six months (mean ranks: <75 y: 38.27; >75 y: 61.75; *p* = 0.001, KWH = −3.357), but not for satisfaction after six months (mean ranks: <75 y: 46.95; >75 y: 34.97; *p* = 0.57, KWH = −1.903). A closer investigation of the age groups divided into CR and PS arthroplasty for under 75 years old and over 75 year old patients showed a significant difference in the range of motion and flexion/extension deficits after six months (for more details see Table 7).

## 4. Discussion

In this prospective study of 123 patients undergoing total knee arthroplasty (TKA), we found that posterior-stabilized (PS) prostheses yielded significantly better outcomes than cruciate-retaining (CR) designs in several domains at six-month follow-up. Specifically, PS-TKA was associated with a shorter operation time, greater postoperative range of motion, and a lower flexion deficit compared with CR-TKA. While no significant differences in patient satisfaction, quality of life, or complication rates were observed between the two implant types, subgroup analyses revealed that age and gender influenced the outcomes: elderly patients (>75 years) with PS-TKA and PS males achieved superior range of motion and lower flexion deficits. Overall, both implant designs improved pain, function, and alignment, but PS-TKA demonstrated advantages in early postoperative mobility, particularly among older patients. Interestingly, a limited range of motion appeared to have had hardly any effect on patient satisfaction. These findings align with recent randomized and cohort studies. Tille et al. (2024) [18] found that PS-TKA achieved better flexion (median 120° vs. 115°, *p* = 0.017) and overall ROM, but patient-reported outcomes (PROMs) and satisfaction were similar between the PS and CR groups at two years. Further studies confirmed these results [18,19,20]. On the other hand, several investigations showed superior outcomes in the Knee Injury and Osteoarthritis Outcome Score (KOOS) or for the general function of the knee for PS groups [6,21]. In this survey, no differences between the prosthesis types were found regarding the postoperative axis. Patient satisfaction, quality of life, and WOMAC scores also showed no significant differences. The same results were observed for patients with high degrees of correction in comparison to patients with lesser axial corrections. A ten-year follow-up study from Park et al. (2018) showed better outcomes for patients with a mechanical alignment ± 3 degrees [22]. When considering parameters from patients with severe malalignment prior to surgery, positive outcomes were still evident regardless of the postoperative axial position [23]. Ultimately, pain appears to be the far more significant factor. As expected, patients showed greater satisfaction, improved quality of life, and lower WOMAC scores at the 6-month follow-up. When looking at preoperative pain perception, quality of life, and WOMAC scores, it was shown that patients with low quality of life scores and high WOMAC scores reported significantly higher pain levels after six months. However, patient satisfaction was not significantly better in patients with low pain scores. The existing literature also shows similar results and identifies increased preoperative anxiety regarding pain or the psychological exaggeration of postoperative pain as additional factors contributing to poorer functional outcomes [24,25]. Surprisingly, the operation times for PS-TKAs were also significantly shorter than those for CR-TKAs. This could have had an influence on the differences in range of motion. Other variables that influenced the outcome after TKA were gender and age. The results of this study showed that male and female patients with PS-TKA had a better range of motion and a lower flexion deficit after six months. In a multicenter study with a 5-year follow-up, Ayers et al. (2024) [11] showed no significant gender differences in functional outcome. Despite a poorer preoperative baseline, women demonstrated a better ability to rehabilitate [11]. Further meta-analyses in the literature reported similar results between gender for complications, revision rates, or pain [26,27]. PS-TKA proved to be the most successful for patients over 75 years of age in terms of the ROM achieved and the lowest flexion deficit on average. Although the ROM showed very good values after six months in patients over 75 years of age, the postoperative quality of life was decreased. A significantly higher test result in the WOMAC score in older patients confirmed these findings. There was no difference between the age groups in terms of postoperative satisfaction. Why older patients perceive their quality of life as more limited despite good functional results needs to be further investigated and explored in greater depth. Other environmental factors in the home environment, loneliness, and a possibly different approach to pain perception may play a role here. A multicenter study of Ayers et al. (2023) [28] found significantly worse pain for young patients preoperatively, but more postoperative pain in elderly patients.

Other studies showed poorer outcome values for revisions and functionality for patients younger than 50 years and better PROM values for patients over 70 years with primary implantation of a TKA [28,29,30].

While our findings suggest advantages for PS designs in ROM and flexion deficit, several limitations warrant caution in interpretation. The substantial attrition (28% loss to follow-up) reduces the power to detect clinically meaningful differences, particularly in age- and gender-stratified analyses. Our single-center design, while ensuring procedural consistency, limits extrapolation to institutions using different implant systems or rehabilitation protocols. Furthermore, the non-randomized allocation may have introduced potential selection bias, as surgeons might have preferentially selected CR designs for patients with better-preserved native kinematics or lower functional demands. The 6-month follow-up of this study can provide early insights into the postoperative outcomes and initial implant function but does not capture potential late complications, implant failures, or the true longevity of the devices. Durability assessments would require longer-term data to evaluate whether implants maintain their integrity, function, and safety over time. Therefore, at least 1–5 years of follow-up will be necessary to observe whether the early benefits persist or if issues such as wear, loosening, or delayed adverse events might emerge. In addition, the limited sample size may also influence the results of this investigation. Surgeons should consider that both PS and CR designs offer substantial improvements in pain, function, and alignment at six months, with PS-TKA demonstrating advantages in early postoperative mobility, particularly for elderly patients. Implant selection should be individualized, taking into account patient age, gender, and functional demands with a preference for PS designs in older or less mobile and male patients to enhance early rehabilitation. Researchers are encouraged to conduct larger, multicenter, and long-term studies to validate these findings. Future research should also investigate why elderly patients report a lower quality of life despite good functional results and aim to identify strategies to optimize patient-reported outcomes across diverse populations.

## 5. Conclusions

Both cruciate-retaining and posterior-stabilized total knee arthroplasty designs provided substantial improvements in pain, function, and alignment at six months postoperatively, with no significant differences in patient satisfaction, quality of life, or complication rates. Posterior-stabilized knees offer a superior range of motion and reduced flexion deficits in the short-term, particularly benefiting elderly patients and those with greater preoperative mobility limitations. No significant gender-based differences in outcomes were observed after six months. Elderly patients reported less quality of life, more pain, and difficulties in everyday life.

In clinical practice, the choice between CR and PS designs should be individualized, especially apart from the posterior cruciate ligament integrity, where patient age, functional demands, and surgeon experience need to be considered. Both designs remain reliable options, with PS knees potentially favoring early functional gains. Particularly for older patients, the classic conventional design with neutral alignment could be the better choice to enhance early rehabilitation and early everyday independence. Further large-scale, long-term randomized studies are warranted to clarify the optimal implant selection for diverse patient populations.

## Figures and Tables

**Table 1:** Patient demographics; (d) = days.

Demographics	Hospitalization	Follow-Up
**Age**	67.94 ± 10.14 years	69.01 ± 8.9 years
**Men**	34.1% (42 of 123)	30.7% (27 of 88)
**Women**	65.9% (81 of 123)	69.3% (61 of 88)
**BMI**	32.09 ± 6.88 kg/m^2^	31.92 ± 6.50 kg/m^2^
**ASA score**	1.95 ± 0.68	1.94 ± 0.73
**Pre-diseases (median)**	2.00	2.50

**Table 2:** The use of walking aids preoperatively and after six months.

	None	Forearm Crutches	Walker	Wheelchair	(*p*); phi
Preoperative					
PS n = 69	73.9%	11.59%	13.0%	1.4%	0.891; 0.102
CR n = 51	80.4%	9.8%	9.8%	-	
After six months					
PS n = 52	63.4%	19.2%	17.3%	-	1.000; 0.036
CR n = 34	64.7%	20.5%	14.7%	-	

**Table 3:** Walking distance preoperatively and after six months.

	Unlimited	Up to 500 m	Under 500 m	Room Mobility	Immobile	(*p*); phi
Preoperative						
PS n = 70	5.7%	61.3%	30.0%	1.5%	1.5%	0.692; 0.145
CR n = 51	5.8%	58.8%	29.4%	5.8%	-	
After six months						
PS n = 52	11.5%	21.2%	67.3%	-	-	0.942; 0.043
CR n = 34	11.8%	17.6%	70.6%	-	-	

**Table 4:** Range of motion, extension deficits, flexion deficits, and leg axis pre- and postoperative and after six months; * = statistically significant. Indication of the range of motion, deficits and axis in degrees (°).

	PS-TKA (±SD)	CR-TKA (±SD)	(*p*), 95%CI
Range of motion ° preoperative	100.29 ± 17.23	100.10 ± 18.01	0.954, [−6.270/−6.645]
Range of motion ° during rehab	76.31 ± 30.08	69.83 ± 40.75	0.492, [−12.218/−25.171]
Range of motion ° after 6 months	105.19 ± 15.56	93.29 ± 15.09	* 0.001, [5.223/18.591]
Extension deficits ° preoperative	5.36 ± 6.95	4.41 ± 5.8	0.515, [−7.094/3.578]
Extension deficits ° during rehab	2.74 ± 9.76	4.17 ± 10.75	0.559, [−6.285/−3.428]
Extension deficits ° after 6 months	3.29 ± 6.62	5.14 ± 8.44	0.255, [−5.074/1.306]
Flexion deficits ° preoperative	23.93 ± 14.03	25.69 ± 15.42	0.404, [−7.094/3.578]
Flexion deficits ° during rehab	32.14 ± 23.89	28.00 ± 23.25	0.466, [−7.123/15.409]
Flexion deficits ° after 6 months	23.56 ± 19.73	37.57 ± 23.33	* 0.003, [**−23.252/−4.776**]
Leg axis ° (α) preoperative	8.04 ± 4.52	6.99 ± 4.49	0.310, [−0.9990/3.1024]
Leg axis ° (α) postoperative	2.92 ± 2.17	2.57 ± 2.13	0.479, [−0.6288/1.3276]
Leg axis ° (α) after six months	2.80 ± 2.15	3.05 ± 2.11	0.729, [−1.2257/0.7363]

**Table 5:** Clinical data; NRS: numeric pain scale; The NRS has a range of 1–10, where 1 is the “lowest pain” and 10 is the “highest pain”; mov = movement; mo = months; dpr = degree of pain reduction; η^2^p = partial eta square; * = statistically significant.

	PS-TKA (±SD)	CR-TKA (±SD)	(*p*), 95%CI
Time of surgery (min)	83.31 ± 25.65	95.26 ± 24.61	[* **0.011, −0.837/−0.108**]
NRS pre-surgery rest	3.81 ± 2.23	4.29 ± 2.61	[0.147, −1.502/0.361]
NRS pre-surgery mov	7.44 ± 1.54	7.53 ± 1.75	[0.808, −0.748/0.579]
NRS post-surgery (6 mo) rest	1.08 ± 2.06	0.97 ± 1.99	[0.813, −0.780/0.991]
NRS post-surgery (6 mo) mov	2.48 ± 2.50	2.46 ± 2.99	[0.968, −1.154/1.201]
NRS (dpr) pre-post (6 mo) surgery	2.51 ± 2.89	3.21 ± 2.48	[0.329, −0.768/0.258]
**ANCOVA analysis**			(*p*), η^2^p
NRS pre-surgery rest	See above (s. a.)		0.367, [0.009]
NRS pre-surgery mov	s. a.		0.801, [0.001]
NRS post-surgery (6 mo) rest	s. a.		0.813, [0.001]
NRS post-surgery (6 mo) mov	s. a.		0.968, [0.000]
NRS (dpr) pre-post (6 mo) surgery	s. a.		0.329, [0.015]

**Table 6:** Gender analysis after CR- and PS-TKA; OP = operation, ROM = range of motion, 6 m = six months; *p* and 95%CI between the groups; * = statistically significant (Bonferroni corrected). Indication of the range of motion and deficits in degrees (°).

	PS-Male	CR-Male	PS-Female	CR-Female	(*p*), 95%CI
Total ROM °	101.33 ± 16.39	102.27 ± 20.66	99.50 ± 18.00	99.50 ± 17.46	0.940, [0.000/0.015]
Flexion deficit °	23.33 ± 12.95	22.27 ± 14.89	24.38 ± 14.94	26.63 ± 15.63	0.738, [0.000/0.047]
Extension deficit °	5.33 ± 6.81	6.36 ± 7.10	5.38 ± 7.19	3.88 ± 5.48	0.605, [0.000/0.061]
Total ROM ° 6 m	108.75 ± 16.45	89.29 ± 15.66	102.97 ± 14.80	94.29 ± 15.07	* 0.003, [0.21/0.271]
Flexion deficit ° 6 m	17.50 ± 14.46	36.43 ± 13.13	27.34 ± 21.77	37.86 ± 25.43	0.011, [0.008/0.239]
Ext. deficit ° 6 m	3.75 ± 6.43	4.29 ± 5.34	3.00 ± 6.81	5.36 ± 9.12	0.678, [0.000/0.073]

**Table 7:** Age groups: m = months; * = statistically significant (Bonferroni corrected). ROM = range of motion; Indication of the range of motion and deficits in degrees (°).

	PS- < 75	CR- < 75	PS- > 75	CR- > 75	(*p*), 95%CI
Total ROM °	101.45 ± 17.18	101.07 ± 18.66	96.00 ± 17.34	95.56 ± 14.67	0.602, [0.000/0.061]
Flexion deficit °	23.18 ± 14.39	24.88 ± 16.21	26.67 ± 12.91	29.44 ± 11.02	0.615, [0.000/0.060]
Extension deficit °	4.82 ± 6.30	4.29 ± 5.79	7.33 ± 9.03	5.00 ± 6.61	0.489, [0.000/0.072]
Total ROM ° 6 m	105.13 ± 15.10	94.50 ± 15.98	105.38 ± 17.49	86.00 ± 4.18	0.005, [0.016/0.259]
Flexion deficit ° 6 m	24.74 ± 20.80	33.83 ± 20.83	20.00 ± 16.33	50 ± 27.86	* 0.001, [**0.000/0.086**]
Extension deficit ° 6 m	2.85 ± 6.35	5.33 ± 8.99	4.62 ± 7.48	4.00 ± 4.18	0.580, [0.031/0.292]

## Data Availability

The datasets used and analyzed during the current study are available from the corresponding author on reasonable request.

## Abbreviations

ASA	The American Society of Anesthesiology
BMI	Body mass index
CRP	C reactive protein
CR	Cruciate retaining
Hb	Hemoglobin
KOOS	Knee Injury and Osteoarthritis Outcome Score
KWH	Kruskal–Wallis H
η^2^p	Partial eta square
PS	Posterior stabilized
PROMs	Patient reported outcome measures
QoL	Quality of life
ROM	Range of motion
TKA	Total knee arthroplasty
WOMAC	Western Ontario and McMaster Universities Osteoarthritis Index
